# Children's Mental Health Visits to the Emergency Department: Factors Affecting Wait Times and Length of Stay

**DOI:** 10.1155/2014/897904

**Published:** 2014-01-19

**Authors:** Amanda S. Newton, Sachin Rathee, Simran Grewal, Nadia Dow, Rhonda J. Rosychuk

**Affiliations:** ^1^Faculty of Medicine & Dentistry, University of Alberta, Edmonton Clinic Health Academy (ECHA), 11405-87 Avenue, Room 3-526, Edmonton, AB, Canada T6G 1C9; ^2^Faculty of Medicine & Dentistry, University of Alberta, WC Mackenzie Health Sciences Centre, Edmonton, AB, Canada T6G 2R7; ^3^Faculty of Medicine & Dentistry, University of Alberta, Edmonton Clinic Health Academy (ECHA), 11405-87 Avenue, Room 3-582B, Edmonton, AB, Canada T6G 1C9; ^4^Faculty of Medicine & Dentistry, University of Alberta, Edmonton Clinic Health Academy (ECHA), 11405-87 Avenue, Room 3-582, Edmonton, AB, Canada T6G 1C9; ^5^Faculty of Medicine & Dentistry, University of Alberta, Edmonton Clinic Health Academy (ECHA), 11405-87 Avenue, Room 3-524, Edmonton, AB, Canada T6G 1C9

## Abstract

*Objective*. This study explores the association of patient and emergency department (ED) mental health visit characteristics with wait time and length of stay (LOS). *Methods*. We examined data from 580 ED mental health visits made to two urban EDs by children aged ≤18 years from April 1, 2004, to March 31, 2006. Logistic regressions identified characteristics associated with wait time and LOS using hazard ratios (HR) with 95% confidence intervals (CIs). *Results*. Sex (male: HR = 1.48, 95% CI = 1.20–1.84), ED type (pediatric ED: HR = 5.91, 95% CI = 4.16–8.39), and triage level (Canadian Triage and Acuity Scale (CTAS) 2: HR = 3.62, 95% CI = 2.24–5.85) were statistically significant predictors of wait time. ED type (pediatric ED: HR = 1.71, 95% CI = 1.18–2.46), triage level (CTAS 5: HR = 2.00, 95% CI = 1.15–3.48), number of consultations (HR = 0.46, 95% CI = 0.31–0.69), and number of laboratory investigations (HR = 0.75, 95% CI = 0.66–0.85) predicted LOS. *Conclusions*. Based on our results, quality improvement initiatives to reduce ED waits and LOS for pediatric mental health visits may consider monitoring triage processes and the availability, access, and/or time to receipt of specialty consultations.

## 1. Introduction

In recent years, there has been considerable documentation of increases in visits by children to the emergency department (ED) for crisis mental health care [1–9]. Studies show that more parents are seeking care for their children in hospital EDs to treat acute emergencies [2, 4–6, 10, 11], request guidance for at-home child management [12], and gain access to health care resources [12]. The current health care system, however, does not meet the needs of families in the emergency care setting [3, 4, 13]. Many children do not receive comprehensive treatment for pediatric mental health visits and are discharged without adequate recommendations for follow-up care [14]. There is also increasing evidence for long waits for care and lengths of ED stay (LOS) [6, 8, 9, 15–19].

A stance among a number of studies is that wait times and LOS are important measures of treatment timeliness and patient safety [20–25]. A number of organizational and patient characteristics have been linked to wait time and LOS and serve to highlight the multifactorial nature of improving ED performance. Longer ED wait times for patients of all ages have been associated with a higher triage level (which denotes a lower level of urgency for the presenting complaint), an increased patient census (ED occupancy), urban-based EDs, day of arrival (Sunday, Monday, or Wednesday), and arrival by “walk-in” (versus ambulance) [26–31]. ED wait times have also been associated with race, ethnicity, and sex [30–35]—sociodemographic disparities that raise issues of access to timely care in terms of issues of bias/discrimination, language barriers, and ED cultural competence. A longer LOS for pediatric visits has also been associated with night shift and early morning arrivals, admitted patients, presentation during the winter season, higher triage acuity, and treatment that includes diagnostic testing and subspecialty consultations [34, 36]. Intentional self-injury, age 6–13 years, use of laboratory testing, hospital location, and patient transfer have also been associated with extended ED stays for pediatric mental health visits [17], while limited staff availability for psychiatric assessments, clinical instability, and limited bed availability have been associated with longer ED LOS for adult mental health visits [37]. In total, this body of literature raises important questions for pediatric emergency mental health care including how to ensure treatment timeliness and quality of care. We analyzed data from a sample of pediatric mental health visits to a general and a pediatric ED over a two-year period to explore organizational and patient characteristics associated with longer wait times and LOS.

## 2. Materials and Methods

### 2.1. Study Population Variables of Interest

We examined data from a previously conducted medical record and administrative database review of a representative sample of pediatric (≤18 years) mental health visits to a general and a pediatric ED, both academic tertiary care centers. Using a proportional allocation stratified random sampling design, 580 ED visits were reviewed from the 2 sites (*n* = 164 for pediatric ED; *n* = 416 for general ED). The site samples ensured that data were representative of mental health emergency visits at each ED location. Characteristics of the EDs and the study protocol are detailed elsewhere [14, 38]. ED visits by children were made between April 1, 2004, and March 31, 2006, for mental illness, substance abuse, or intentional self-harm (International Statistical Classification of Diseases (ICD) codes) [39].

Clinical data included in this study were health care visit and demographic variables of interest. Health care visit data included the type of ED visited (general versus pediatric), the presenting complaint (to indicate primary reason for mental health visit), and ED accompaniment and mode of arrival. Other ED visit information included the time of week (Tuesday to Thursday, Friday to Monday) and season (spring, summer, autumn, or winter) for the visit. We also included triage level data coded according to the Canadian Triage and Acuity Scale (CTAS; CTAS 1 = Resuscitation, CTAS 2 = Emergent, CTAS 3 = Urgent, CTAS 4 = Semi-Urgent, CTAS 5 = Non-Urgent) [40, 41]. This triage level is assigned at ED presentation to establish treatment priority based on the severity of the child's condition. We were also interested in whether a child received a mental health consultation, the number of consults and diagnostic tests (investigations) documented for the ED visit, and the recorded disposition for the visit. Documented ED registration, triage, assessment (physician and nurse), and discharge dates and times were also reviewed to calculate the wait time and LOS for each ED visit. Demographic data included age, sex, the presence of medical or psychiatric comorbidities (yes/no), and socioeconomicstatus (SES) based on median household income [42]. This study was approved by the Health Research Ethics Board of the University of Alberta (Edmonton, Alberta).

### 2.2. Outcome Variables

Two time-to-event outcome variables defined two patient subsets. For patients who had a disposition other than “left without being seen” (admitted, discharged, transferred, or unknown), the ED wait time for the visit was defined as the time of triage to the first time assessed by a health care provider (nurse or physician, whichever time came first). LOS for the visit was defined as the time of triage to the time of discharge from the ED for the subset of patients who were discharged or admitted to hospital.

### 2.3. Statistical Analysis

Data were summarized by frequencies and percentages or median and interquartile range (IQR). If the triage time was missing for the ED visit, the registration time was used in the calculation of the outcome variables. Missing, negative, or implausible wait times were replaced with median times for the respective triage level and censored. The median wait time for triage level 3 was used as the censoring time for patients with missing triage. For LOS, missing, negative, or implausible LOS times were replaced with median times for the respective diagnosis and censored [23].

Kaplan-Meier curves display outcome variables and separate bivariable and multivariable Cox proportional hazards (PH) models (with a random effect for patients) were developed to investigate predictors for each outcome variable. Based on previous studies, variables hypothesized to predict longer ED wait times for pediatric mental health visits were those that occurred over the weekend/early week (Friday to Monday) and had less urgent triage levels and were made by patients with a lower SES and who arrived by “walk-in” (versus ambulance) [26–34]. We also hypothesized that lower SES would predict a longer ED wait time based on bias/discrimination findings from other studies [30–35] and that youth who were not accompanied by a guardian or parent (e.g., arrival alone or with friends) waited longer because of the absence of adult advocacy and communication limitations. Variables hypothesized to predict a longer ED LOS were those visits that occurred over the weekend/early week (Friday to Monday), had more urgent triage levels, involved mental health consultations and other consultations/laboratory investigations, and resulted in the patient being admitted [17, 34, 36]. We also hypothesized that those visits made to the general ED would result in a longer LOS [37] as this hospital had in-house psychiatric service compared to the pediatric ED and would likely involve more assessment/care [14]. An interaction term (ED type and number of investigations) was added to the LOS model to account for known practice variation between EDs [14]. Models were examined for the PH assumption [43]. Hazard ratios (HR) and 95% confidence intervals (CIs) are reported. Statistical software (S-PLUS Version 8.1.1 for Linux, TIBCO Software Inc., Palo Alto, CA, 2008) was used for data analysis; *P* < 0.05 was considered statistically significant.

## 3. Results

Of the 580 mental health ED presentations in this study, visits were made by 551 distinct children and youth. The majority of ED visits were made by females (*n* = 326, 56.2%) and youth aged 13–18 years (*n* = 494, 85.2%) and children aged 6–12 years (*n* = 81, 14.0%). ED visits were represented across all SES groups; however, the majority of visits involved children and youth from median annual family incomes of $50,000–$69,999 (*n* = 322, 55.5%) and $30,000–$49,999 (*n* = 91, 15.7%). Only 2.6% of visits were made by children and youth from a median family income <$30,000 (*n* = 15). Visits were made equitably across seasons (spring: 25.5%, summer: 22.1%, autumn: 25.5%; winter: 26.9%).

Children and youth were most often accompanied by a parent or guardian (*n* = 316, 54.4%) with “walk-in” (*n* = 268, 46.2%) as the most common method of arrival (Table 1). The majority of presentations were assigned a triage level of CTAS 3 (*n* = 271, 46.7%) and CTAS 4 (*n* = 215, 37.1%) at presentation. Mental and behavioural disorders secondary to substance abuse (*n* = 161, 27.8%) and behavioural/emotional disorders and syndromes (*n* = 122, 21.0%) were the most frequent main ambulatory diagnoses. Comorbidities were documented for 249 children and youth (42.9%). Most children and youth did not receive a mental health consultation (*n* = 399, 68.8%). The majority of children and youth presenting for a mental health crisis did not receive any other specialty/subspecialty consultation (*n* = 365, 62.9%) during their ED visit and laboratory investigations were often not ordered (*n* = 411, 70.9%). Most ED presentations for mental health by children and youth ended in discharge (*n* = 426, 73.4%) (Table 1).

**Figure 1:**
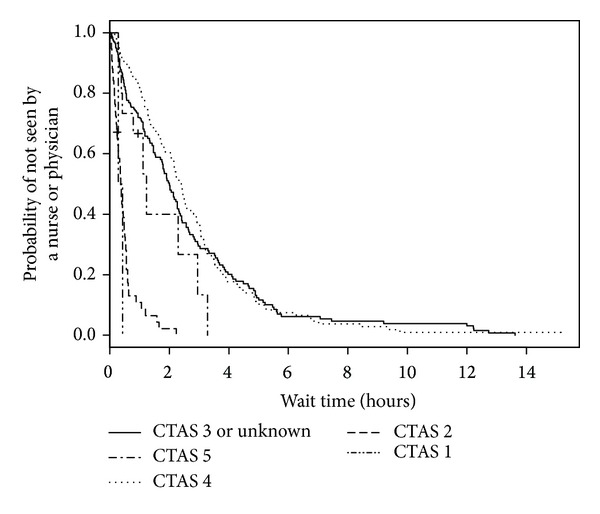
Kaplan-Meier estimates of ED wait time by triage level.

**Figure 2:**
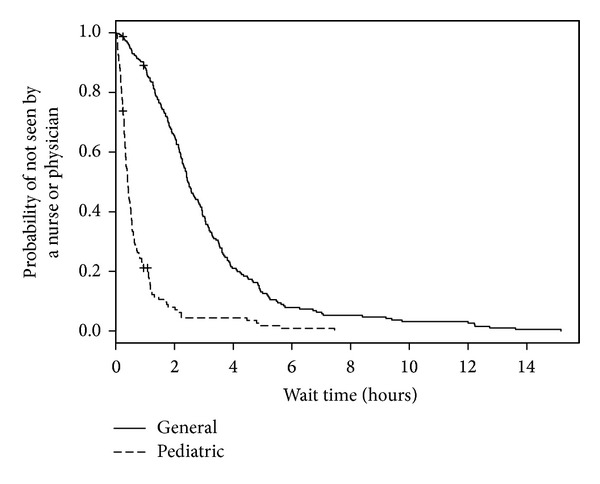
Kaplan-Meier estimates of ED wait time by ED type.

**Figure 3:**
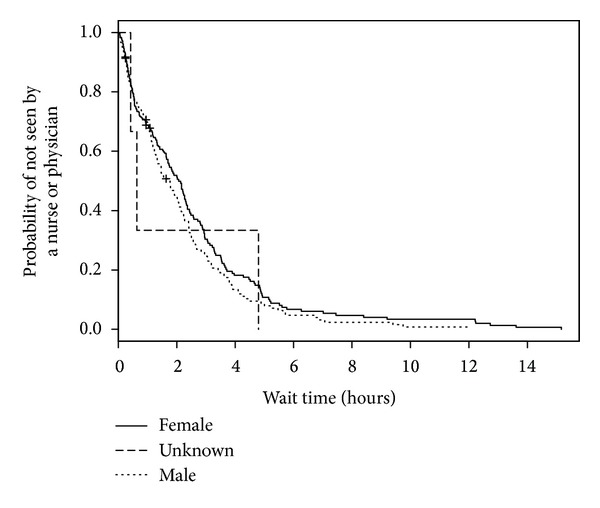
Kaplan-Meier estimates of ED wait time by sex.

We analyzed 558 ED visits (by 531 distinct patients) for modeling predictors of longer wait times. Of these visits, all had a disposition other than “left without being seen” and 1 visit had an unknown triage time. In the medical records for these visits, 3 records did not have recorded sex, 24 records did not have patient accompaniment documented, 23 records did not have mode of ED arrival documented, and 33 records did not have the necessary forward sortation data available to calculate SES. The median wait time for these visits was 1 hour and 5 minutes (IQR: 32 minutes, 1 hour and 50 minutes). When predictor variables were examined separately (Table 2; bivariable model), triage level, ED type, mode of ED arrival, and diagnosis were statistically significant predictors of wait time. When adjusted for other predictors in the multivariable model, sex became statistically significant and only ED type and triage level remained statistically significant (Table 2; multivariable model). As seen in Figure 1, when compared with those children and youth triaged as CTAS 3, children and youth triaged as CTAS 2 had shorter wait times (HR = 3.62, 95% CI = 2.24–5.85). The relatively large CIs reflect small counts for CTAS 1 (*n* = 2). Children and youth who visited a pediatric ED were much more likely to wait less time to see a health care provider (HR = 5.91, 95% CI = 4.16–8.39; Figure 2) than those children and youth who visited a general ED. Shorter wait times were associated with male sex (HR = 1.48, 95% CI = 1.20–1.84; Figure 3). The multivariable model showed some deviation from the assumption of proportional hazards for some of the variables including ED type, CTAS, and diagnosis.

**Figure 4:**
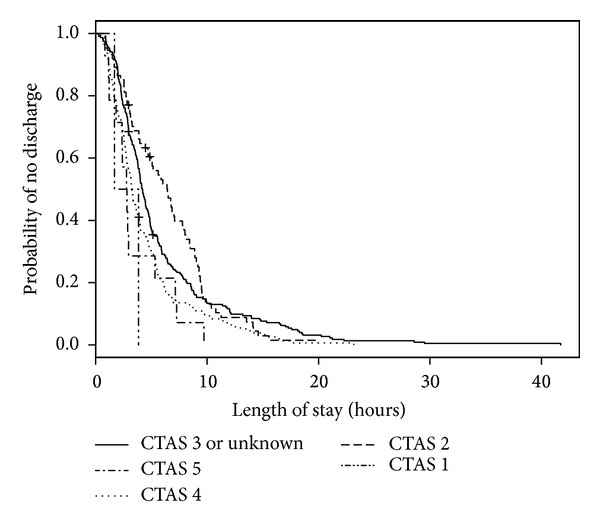
Kaplan-Meier estimates of ED LOS by triage level.

**Figure 5:**
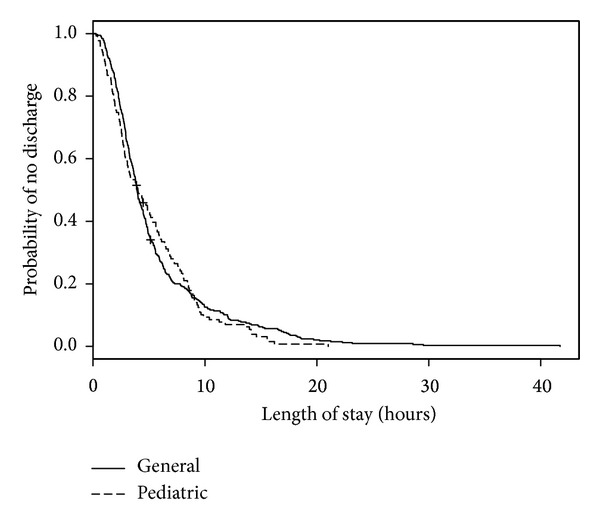
Kaplan-Meier estimates of ED LOS by ED type.

**Figure 6:**
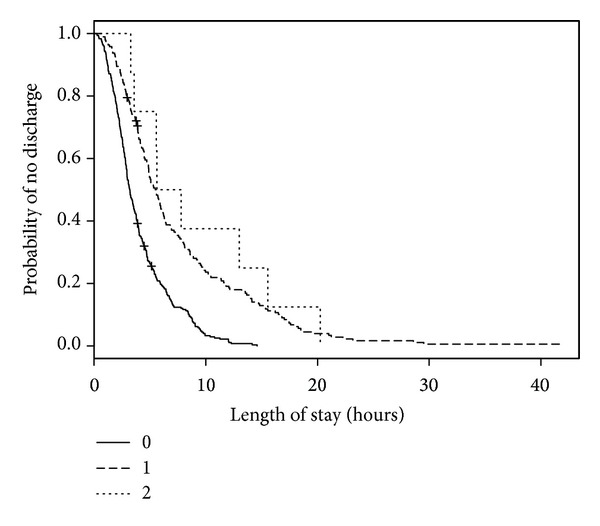
Kaplan-Meier estimates of ED LOS by number of consultations.

**Figure 7:**
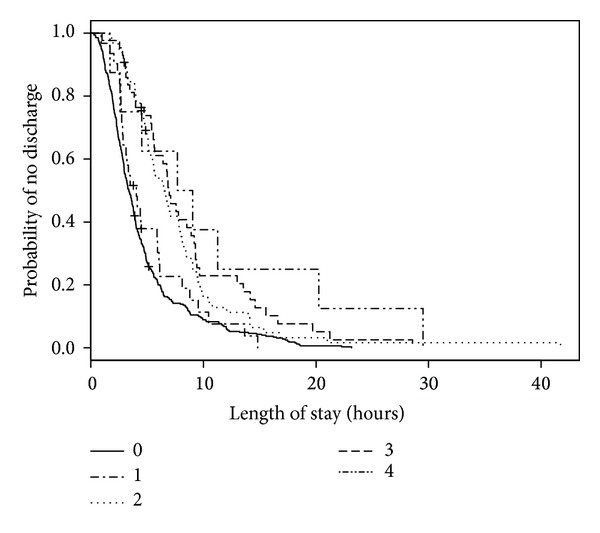
Kaplan-Meier estimates of ED LOS by number of investigations.

We analyzed 501 ED visits with a disposition of either admitted or discharged (by 477 distinct patients) for modeling predictors of longer LOS. Of these visits, 1 visit had an unknown triage time. In the 501 medical records, 2 records were missing sex, 10 did not have patient accompaniment documented, 10 records did not have mode of ED arrival documented, and 27 records did not have the necessary forward sortation data available to calculate SES. The median ED LOS was 3 hours and 53 minutes (IQR: 2 hours and 26 minutes, 6 hours and 24 minutes). When predictor variables were examined separately (Table 3; bivariable model), age, triage level, mental health consultation, disposition, number of consultations, number of laboratory investigations, and diagnosis were statistically significant predictors of LOS. For the multivariable model, an interaction term of ED type by number of laboratory investigations was added to account for known practice variation between EDs. When adjusted for other predictors in the multivariable model, ED type became statistically significant. Triage level, number of consultations, and number of laboratory investigations remained statistically significant (Table 3; multivariable model). When compared with those children and youth triaged as CTAS 3, children and youth triaged as CTAS 5 had a shorter LOS (HR = 2.00, 95% CI = 1.15–3.48; Figure 4). The relatively large CIs reflect small counts for CTAS 1 (*n* = 2). Children and youth who visited a pediatric ED (HR = 1.71, 95% CI = 1.18–2.46; Figure 5) were more likely to experience a shorter LOS than those who visited a general ED. A longer ED LOS for pediatric mental health visits was also associated with the number of consultations (HR = 0.46, 95% CI = 0.31–0.69; Figure 6) and number of laboratory investigations (HR = 0.75, 95% CI = 0.66–0.85; Figure 7) that occurred during the visit. The multivariable model showed some deviation from the assumption of proportional hazards for some of the variables: ED type, number of consults, and number of investigations.

## 4. Discussion

In this study, we explored the association of patient and ED visit characteristics with wait time and LOS for pediatric mental health visits. In our multivariable model, shorter ED wait times were associated with visits to a pediatric ED, children and youth triaged as CTAS 2, and being male, while shorter LOS was associated with visits to a pediatric ED, children and youth triaged as CTAS 5, and fewer consultations and laboratory investigations that occurred during the visit. Based on these results, candidate characteristics for quality improvement initiatives in EDs to reduce waits and LOS for pediatric mental health visits are discussed below.

In this study, it mattered in terms of both wait time and LOS whether a child or youth visited a pediatric or general ED. Consistent with our hypothesis, those visits made to the general ED resulted in a longer LOS. This finding may reflect the psychiatric resources available at the general hospital as previous research has shown that children/youth who visited this ED were more likely to receive mental health consultations and associated care [14]. While a longer LOS due to access to specialized services does not necessarily imply a need for quality improvement, the length of time to accessing such resources in the ED (e.g., wait time for consultations) is of importance and should be tracked by EDs to ensure timely access to assessments and treatment. Other factors that may have affected the longer wait and LOS at the general ED which would be important for addressing treatment timeliness and quality of care, but not assessed in this study, were the mixed patient population (adult and pediatric, emergencies other than mental health) and rates of discharge/admission. These factors have been found to affect health care delivery to children seen in different ED types [25].

Adult, female patients (>18 years) have been found to wait longer for ED assessment and treatment than male patients [31, 36], and it has been proposed for adult patients that differences may be, in part, attributable to a lack of preassessment/treatment testing (e.g., EKG) [31]. The role of sex in influencing ED wait times amongst different ages of children and youth has not been previously documented, and the reasons for this difference seen in our study for pediatric mental health patients remain unclear. Further investigation is important to determine whether a difference in preassessment for these patients is a factor, which may have implications for the utilization of standardized preassessment tools at triage.

While other studies have reported racial and ethnic disparities amongst adult and pediatric ED patients for ED wait time and LOS [30–35], we did not find SES, another type of patient sociodemographic, to be a significant predictor of either time period. In other studies, the influence of insurance status on ED wait times has been mixed with several studies reporting no effect [32, 44] and an Australian study reporting lower SES as a significant predictor of ED wait time [36]. Based on this body of results, it may be that different organizational characteristics (e.g., location of ED, whether mental health services are available in the ED) interact with insurance status and SES to influence ED wait time and LOS. This interaction may be important for future studies interested in developing quality improvement initiatives for ED wait times and LOS.

Day of the week when the ED visit occurred was also not a significant predictor of wait time or LOS as we had hypothesized. While other studies have reported longer times for visits on Sunday, Monday, and Wednesday [27, 28], our findings are similar to Chan et al. [45] who found no difference in day of the week on overall ED LOS. The difference in study patient populations may explain our findings in that adult patients with medical concerns wait until the weekend or those who do not improve over the weekend decide to seek care shortly thereafter, whereas parents caring for a child with mental health needs may be motivated during any day of the week to visit the ED given the caregiving and emotional demands of mental health crises.

In this study, mode of arrival and patient accompaniment were not significant predictors of ED wait time. However, other studies discuss the implications of providing care to an unaccompanied minor [46], and we recommend that those EDs who treat unaccompanied minors in emergency mental health situations use age appropriate language to help elicit information needed to stabilize the crisis, facilitate timely care, and provide a comprehensive referral/discharge plan if needed.

Comparable to other study findings [27, 28, 31, 36], a significant predictor of wait time and LOS in this study was triage level. As expected, mental health visits triaged at the higher acuity CTAS 2 had shorter waits for care. CTAS 1 was also a significant predictor of a shorter LOS, as hypothesized; however, the large confidence interval suggests that further evaluation with a larger sample size would lend confidence to this finding. A longer ED wait time is a known risk factor for a longer LOS [36]. While visits triaged as CTAS 5 in this study had longer recorded wait times, this lower acuity triage level was a significant predictor of a shorter LOS. Less urgent conditions usually require minimal physician/nursing consultation and often only assessment/reassurance, which could lead to more efficient treatment and discharge times [23]. Triage associated initiatives shown to improve timeliness to care that may impact the visits include having a health care provider (i.e., nurse practitioner, physician assistant) in the triage area to perform initial assessments and initiate diagnostic tests [47], general practice clinics adjacent to the ED to divert nonurgent patients [48], and bedside registration/triage which involves the primary care nurse working alongside registration staff at a mobile computer station to log presenting complaints and the initial assessment [49]. These strategies may also improve those mental health visits with a longer LOS (e.g., CTAS 3) by improving time delays caused by waiting for a number of subspecialty consultations and laboratory investigations, which in our study were found to predict a longer LOS.

Of final note, disposition status was not a significant predictor of LOS in our study. Other studies have noted that mental health patients presenting to the ED tend to spend a significant time there for evaluation and disposition due to insufficient psychiatric services and inpatient resources (i.e., available beds for admission) [17, 19]. In some studies, being transferred to another health care facility or being admitted [17, 34] increased the odds of an extended LOS. Variables not evaluated in this study may further explain our finding and be of importance to quality improvement, such as the presence of any quality improvement measures to facilitate discharge and/or the availability of inpatient beds.

## 5. Study Limitations

There are some limitations to this study. The data set was limited by date (March 31, 2006) and thus may not entirely reflect current predictors of ED wait time and LOS.

Other determinants (such as the variable availability of a specialized mental health assessment team or a potential lack of comfort for some physicians in knowing how to manage patients with mental health concerns, thereby preferentially selecting other patients to see first) likely contribute to both ED wait and LOS, but these factors could not be elicited in the medical records we reviewed. Sociodemographic disparities in ED wait and LOS based on race and ethnicity have also been discussed in the literature, but this information was not routinely collected or available in the medical records we reviewed. Further, administrative data such as ED census during the date/time of the mental health visit and the availability of in-patient beds (e.g., boarding time) may also help to explain our findings but were not collected in this study.

## 6. Conclusions

In summary, the results of our multivariable model suggest that, as a first step, quality improvement initiatives in EDs for treatment timeliness and safety for pediatric mental health visits include the monitoring of triage processes, and the availability, access, and/or time to involve specialty consultations. Further work is needed to better understand the role of sex in influencing wait time for pediatric mental health patients as is the influence of ED-wide quality improvement measures to facilitate throughput such as discharge and/or the availability of in-patient beds.

## Figures and Tables

**Table 1:** Visit characteristics (*n* = 580), *n* (%).

ED type	
General	416 (71.7)
Pediatric	164 (28.3)
Mode of arrival	
Medical transport	267 (46.0)
Police	15 (2.6)
Walk-in	268 (46.2)
Unknown	30 (5.2)
Patient accompaniment	
Alone	9 (1.6)
Emergency Medical Services (EMS) worker	213 (36.7)
Parent/guardian	316 (54.4)
Friend	11 (1.9)
Unknown	31 (5.3)
Triage level	
CTAS 1	2 (0.3)
CTAS 2	76 (13.1)
CTAS 3	271 (46.7)
CTAS 4	215 (37.1)
CTAS 5	15 (2.6)
Unknown	1 (0.2)
Main ambulatory diagnosis	
Anxiety or stress-related disorder	112 (19.3)
Behavioural or emotional disorder/syndrome	122 (21.0)
Intentional self-harm	62 (10.7)
Mental and behavioural disorder secondary to substance abuse	161 (27.8)
Mood disorder	100 (17.2)
Schizophrenia or other psychotic disorder	23 (4.0)
Comorbidity	
None documented	331 (57.1)
Mental health consultation	
No	399 (68.8)
Yes	181 (31.2)
Number of consultations	
0	365 (62.9)
1	206 (35.5)
2	9 (1.6)
Number of investigations	
0	411 (70.9)
1	40 (6.9)
2	76 (13.1)
3	45 (7.8)
4	8 (1.4)
Disposition	
Admitted	75 (12.9)
Discharged	426 (73.4)
Left without being seen/left against medical advice	22 (3.8)
Transferred	21 (3.6)
Unknown	36 (6.2)

**Table 2:** Exploratory models of ED wait time predictors for pediatric mental health visits (*n* = 558).

	*n*	Bivariable model			Multivariable model		
		Hazard ratio	95% CI	*P* value	Hazard ratio	95% CI	*P* value
Age	558	1.03	0.99–1.07	0.210	1.01	0.97–1.06	0.521
Sex							
Female	314	Reference			Reference		
Male	241	1.18	0.96–1.44	0.108	1.48	1.20–1.84	<0.001
Unknown	3	1.26	0.39–4.05	0.699	0.46	0.15–1.41	0.174
Socioeconomic status							
<$30,000	15	0.77	0.49–1.22	0.269	1.05	0.62–1.75	0.867
$30,000–$49,999	89	1.04	0.77–1.42	0.792	0.82	0.59–1.14	0.230
$50,000–$69,999	308	Reference			Reference		
$70,000–$89,999	68	0.91	0.65–1.28	0.590	0.92	0.65–1.31	0.653
>$90,000	45	1.09	0.73–1.62	0.680	1.08	0.68–1.70	0.753
Unknown	33	1.54	0.97–2.45	0.068	1.07	0.71–1.64	0.737
Day of the week							
Tuesday to Thursday	265	Reference			Reference		
Friday to Monday	293	1.19	0.97–1.45	0.098	0.98	0.79–1.23	0.846
Patient accompaniment							
Parent/guardian or unknown	331	Reference			Reference		
EMS, alone, or with friends	227	1.02	0.84–1.25	0.836	1.36	0.95–1.95	0.094
Mode of arrival							
Medical transport/police or unknown	298	Reference			Reference		
Walk-in	260	0.59	0.48–0.72	<0.001	0.86	0.57–1.28	0.455
ED type							
General	398	Reference			Reference		
Pediatric	160	5.39	3.78–7.69	<0.001	5.91	4.16–8.39	<0.001
Triage level							
CTAS 1	2	9.47	5.75–15.58	<0.001	2.32	0.90–6.01	0.082
CTAS 2	76	7.08	4.95–10.12	<0.001	3.62	2.24–5.85	<0.001
CTAS 3 or unknown	264	Reference			Reference		
CTAS 4	201	0.89	0.71–1.11	0.310	0.97	0.74–1.26	0.799
CTAS 5	15	1.64	0.95–2.82	0.076	1.02	0.60–1.72	0.952
Diagnosis							
Mental/behavioural disorder secondary to substance abuse	152	Reference			Reference		
Anxiety/stress-related disorder	109	0.42	0.31–0.58	<0.001	0.82	0.50–1.32	0.405
Intentional self-harm	61	0.50	0.34–0.75	<0.001	1.15	0.73–1.79	0.550
Mood disorder	97	0.39	0.27–0.56	<0.001	0.97	0.64–1.47	0.889
Schizophrenia or other psychotic disorder	20	0.42	0.25–0.69	<0.001	0.84	0.52–1.34	0.456
Behavioural or emotional disorder/syndrome	119	0.40	0.29–0.56	<0.001	0.97	0.63–1.49	0.898

**Table 3:** Exploratory models of ED LOS predictors for pediatric mental health visits (*n* = 501).

	*n*	Bivariable model			Multivariable model		
		Hazard ratio	95% CI	*P* value	Hazard ratio	95% CI	*P* value
Age	501	0.93	0.90–0.97	<0.001	0.96	0.92–1.01	0.079
Sex							
Female	278	Reference			Reference		
Male	221	0.93	0.77–1.11	0.412	0.88	0.72–1.07	0.210
Unknown	2	1.35	0.50–3.60	0.554	1.77	1.23–2.55	0.002
Day of the week							
Tuesday to Thursday	245	Reference			Reference		
Friday to Monday	256	1.02	0.86–1.22	0.825	1.05	0.87–1.27	0.624
ED type							
General	366	Reference			Reference		
Pediatric	135	1.06	0.88–1.28	0.551	1.71	1.18–2.46	0.004
Triage level							
CTAS 1	2	2.87	1.14–7.21	0.025	4.96	1.57–15.68	0.006
CTAS 2	74	0.86	0.69–1.07	0.181	1.13	0.82–1.56	0.441
CTAS 3 or unknown	238	Reference			Reference		
CTAS 4	173	1.40	1.13–1.73	0.002	1.24	0.99–1.56	0.061
CTAS 5	14	1.81	1.04–3.16	0.037	2.00	1.15–3.48	0.015
Mental health consultation							
No consultation	332	Reference			Reference		
Consultation	169	0.48	0.40–0.59	<0.001	0.93	0.60–1.42	0.722
Disposition							
Discharged	426	Reference			Reference		
Admitted	75	0.67	0.53–0.84	<0.001	0.99	0.77–1.28	0.961
Number of consultations	501	0.45	0.38–0.54	<0.001	0.46	0.31–0.69	<0.001
Number of laboratory investigations	501	0.75	0.70–0.81	<0.001	0.75	0.66–0.85	<0.001
Pediatric ED, number of investigations*	501				0.88	0.73–1.07	0.197
Diagnosis							
Mental/behavioural disorder secondary to substance abuse	129	Reference			Reference		
Anxiety or stress-related disorder	102	1.66	1.27–2.17	<0.001	1.20	0.83–1.74	0.322
Intentional self-harm	54	0.91	0.63–1.31	0.599	1.04	0.70–1.55	0.854
Mood disorder	87	1.06	0.83–1.35	0.637	1.10	0.77–1.58	0.602
Schizophrenia or other psychotic disorder	17	0.83	0.59–1.17	0.295	0.88	0.54–1.44	0.621
Behavioural or emotional disorder/syndrome	112	1.28	0.98–1.66	0.068	1.13	0.79–1.62	0.506

*Interaction with number of investigations.
